# Sensitivity and Specificity of Preschool Vision Screening in Iran

**Published:** 2017-02

**Authors:** Hassan HASHEMI, Abbasali YEKTA, Ebrahim JAFARZADEHPUR, Hadi OSTADIMOGHADDAM, Amir ASHARLOUS, Payam NABOVATI, Mehdi KHABAZKHOOB

**Affiliations:** 1. Noor Research Center for Ophthalmic Epidemiology, Noor Eye Hospital, Tehran, Iran; 2. Dept. of Optometry, School of Paramedical Sciences, Mashhad University of Medical Sciences, Mashhad, Iran; 3. Dept. of Optometry, Iran University of Medical Sciences, Tehran, Iran; 4. Noor Ophthalmology Research Center, Noor Eye Hospital, Tehran, Iran; 5. Refractive Errors Research Center, School of Paramedical Sciences, Mashhad University of Medical Sciences, Mashhad, Iran; 6. Dept. of Medical Surgical Nursing, School of Nursing and Midwifery, Shahid Beheshti University of Medical Sciences, Tehran, Iran

**Keywords:** Vision screening, Amblyopia, Schoolchildren, Sensitivity, Specificity, Iran

## Abstract

**Background:**

To determine the sensitivity, specificity, positive predictive value (PPV), and negative predictive value (NPV) of the National Vision Screening Program for 7 yr old children in Iran.

**Methods:**

In this cross-sectional study, eight cities in Iran were selected through multistage cluster sampling. Selected cities were Sari, Birjand, Ardabil, Mashhad, Bandar Abbas, Dezful, Yazd, and Arak, in Iran in 2013. Totally, 4614 schoolchildren were selected, 4106 of which participated in the study. An optometrist at the school site conducted all vision tests. Results were compared against those recorded on each child’s health card. Those with an uncorrected visual acuity worse than 20/25 in at least one eye screened positive for a vision problem.

**Results:**

8.49% [95% confidence interval, 7.65 to 9.39] of the examinees had a vision problem. The sensitivity rate of the school entry screening was 38.15% (95% CI, 33.01 to 43.50) and the specificity rate was 93.11 (95%CI 92.25 to 93.90). The positive and negative predictive values were 33.93 (29.24 to 38.88) and 94.19 (93.39 to 94.93), respectively. Sensitivity and specificity rates did not significantly differ between boys and girls. For the uncorrected visual acuity tested by public health care workers compared to optometrists, the area under the ROC surface was 0.741 (*P*<0.001). The best-associated criterion was an uncorrected visual acuity more than 0.05 LogMAR with 67.3% sensitivity and 74.7% specificity.

**Conclusion:**

The validity of the school entry vision screening by health workers is low. To reduce false negative rates, some supplementary examinations such as refraction and near visual acuity measurements as well as further training of screeners should be considered.

## Introduction

Totally, 19 million children have some types of vision disorder that are the fourth most common disability among children in the US (1–4).

Conditions such as refractive errors, strabismus, and amblyopia are major causes of vision disorders. While, refractive errors are more common than amblyopia during childhood, the latter can lead to worse consequences in vision and performance (5). In light of the importance of the issue, many countries have dedicated vision screening programs to identify vision problems in children.

Timely identification and treatment of amblyopia can eventually prevent the impairment of vision and learning, and help avoid long-term complications such as their impact on the quality of life (6). Children’s vision screening programs in different countries, such as the Great Britain and the United States, are conducted by teachers, nurses, or simply trained health care personnel using various measurement techniques (7, 8). Cases of amblyopia were originally identified based on visual acuity testing, however, since most 5 yr old children do not cooperate for reading letters and numbers, today objective methods are used as well (9, 10).

Children are also being screened at younger ages; early programs focused on preschool children only while some programs include them as early as 3 yr of age. This approach has caused a 60% reduction in the prevalence of amblyopia (11). The sensitivity rates of screening techniques in different countries range from 25% up to more than 95% (7). This fact highlights the important role of measurement technique and personnel in the validity of screening programs and identifying cases of amblyopia.

In Iran, the National Vision Screening Program for children was established in 1996 (7, 12). In this program, all children are once tested between the ages of 3 to 6 yr before entering preschool, and for cases with amblyopia, parents are advised to seek medical consultation. A second screening for amblyopia is done as they enter elementary school at the age of 6 or 7 yr. A health care worker who tests uncorrected vision using the Snellen chart at 6 meters does this. Since no other screenings are done thereafter, this is the most important and last chance for identifying and treating cases of amblyopia.

In light of the importance of the National Vision Screening Program, this study aimed to address the following: 1) The validity of school entry vision screening; 2) The impact of gender on the validity of vision screening; and 3) Determining the best visual acuity cut point to achieve the best sensitivity and specificity for vision screening.

## Materials and Methods

The present study was conducted cross-sectional. The target population of the study was 7 yr old urban children throughout Iran. Since 98.9% of 7 yr old children are in the first grade of elementary school, the sampled population was first graders throughout Iran.

### Sampling method

**Fig. 1:**
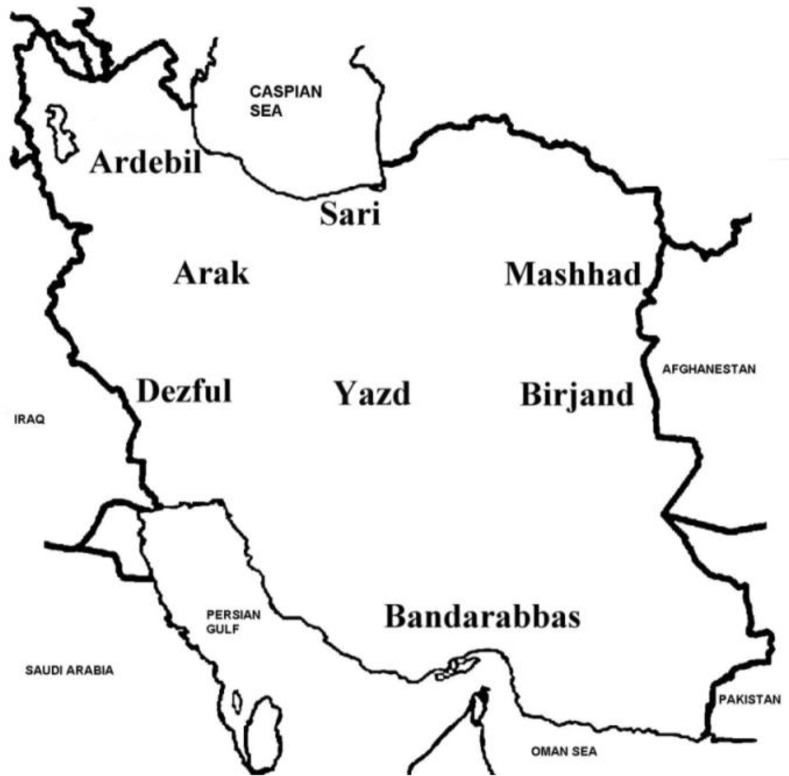
Geographic location of cities selected in this study on the map of Iran

First, eight cities were randomly selected from various geographic regions using multistage cluster sampling (Fig. 1). Selected cities were Sari, Birjand, Ardabil, Mashhad, Bandar Abbas, Dezful, Yazd, and Arak, in Iran in 2013. In each city, first, an equal number of boys’ and girls’ elementary schools were randomly selected, and all their first graders were targeted for vision testing.

Upon identifying sample schools, and making necessary arrangements with the local Office of Education, parent consent forms were signed by parents. Inclusion criteria were having a signed parent consent form and having results of a vision screening done by a health care worker in the past three months.

On the exam day, the examination room in each school was selected by study optometrists to ensure proper lighting and spacing. Children were enrolled into the study based on an alphabetical roster. After extracting demographic information and the vision stated in the health card, the schoolchild proceeded for optometric examinations.

### Examinations

After the initial interview, children entered the exam room and were tested for non-cycloplegic autorefraction by a skilled operator using the TOPCON RM8800 (Topcon Corporation, Tokyo, Japan); results were recorded and printouts were attached to the charts.

For children who had eyeglasses, we tested their visual acuity with their present glasses using tumbling E Snellen charts at 6 meters, and after lensometry with Topcon LM 800 (Topcon Corporation, Tokyo, Japan), we recorded the prescription of the glasses and their prescription date.

In the next stage, all children were tested for uncorrected visual acuity. Then, autorefraction results were refined through retinoscopy using HEINE BETA 200 (HEINE Optotechnik, Germany) and the MSD trial lenses (MSD Meniscus Trial Lenses, Italy). For each child, first, the right and then the left eye were tested. For any child with uncorrected visual acuity worse than 20/25, subjective testing was done and best results with vision correction were recorded. Eventually, all children had cycloplegic refraction testing with the autorefractometer and retinoscopy 35 min after having a drop of cyclopento-late 1% instilled twice, five min apart.

### Definitions

Since the main objective of screening programs is to identify cases of amblyopia, the cut point for uncorrected visual acuity was set at 20/32.

### Statistical Analysis

We used optometrist-measured uncorrected visual acuity test results as the gold standard to assess the validity of the vision tested by the national program. An uncorrected visual acuity equal to or worse than 20/32 in at least one eye was the criterion we used to identify children with a vision problem and to make calculations. The sensitivity, specificity, positive predictive value, and negative predictive value of the vision screening performed by health care workers, and the likelihood ratios (LR) were calculated as demonstrated in Table 1. All results are reported with their 95% confidence interval.

**Table 1:** Definition of sensitivity and specificity of screening test

**The National Vision Screening Program (health care worker)**		**Gold Standard (optometrist) Visual acuity equal to or worse than 20/32 in at least one eye**			
			**Positive**	**Negative**	**total**
	Visual acuity equal to or worse than 20/32 in at least one eye	Positive	A	B	A+B
		Negative	C	D	C+D
		Total	A+C	B+D	n

Sensitivity: A/A+C - Specificity: D /B+D - Positive predictive value: A/A+B - Negative predictive value: D/C+D - Positive likelihood ratio: Sensitivity/100- Specificity - Negative likelihood ratio: 100-Sensitivity/Specificity - Accuracy: (A+D)/A+B+C+D

To determine the optimal cut point, we used receiver operating characteristic (ROC) curves and calculated the area under the curves in the present sample. Since the number of students in each city was not proportionate to the total number of students in that city, the weight of each city in proportion to all cities was taken into account in the analyses. In addition, our study samples were selected using a cluster sampling approach. In this method, the variable of interest can be over-estimated because there is more variance within clusters than between them. To account for clustering, sample size is increased using the design effect adjustment. Therefore, clusters and the design effect must be considered in the analysis and confidence intervals should be calculated after correcting for the design effect.

### Ethical Issues

The Ethics Committee of Arak University of Medical Sciences approved the study protocol, conducted in accord with the tenets of the Helsinki Declaration. All participants` parents signed a written informed consent.

## Results

Seventy elementary schools were selected in eight cities through multistage cluster sampling. Selected students were 4614 children, 4106 of which participated in the study (89% response rate); 51.8% (n=2127) of the participants were boys. Table 2 shows the number of selected students compared to the total number of school-children in each city. Table 3 displays the number of schoolchildren with vision equal to or worse than 20/32 based on screening examinations conducted by health workers and study optometrists. As this two-by-two table demonstrates, 8.49% (95% CI: 7.65 to 9.39) of the schoolchildren had 20/32 or worse uncorrected vision in at least one eye based on optometrists’ tests. The sensitivity and specificity rates of uncorrected vision testing by health workers were 38.15% (95% CI: 33.01 to 43.50) and 93.11% (95% CI: 92.25 to 93.90), respectively. In addition, as stated in Table 3, the positive and negative predictive values were 33.93% (95% CI: 29.24 to 38.88) and 94.19 (95%CI: 93.39 to 94.93), respectively.

**Table 2:** Sample size and total population of schoolchildren in each city

**Location**	**Participants**	**Total population of schoolchildren in 2013**	**Weighing for sampling**
Dezful	498	59980	2.20
Bandar Abbas	499	13263	0.49
Ardebill	533	13164	0.45
Birjand	553	7080	0.23
Sari	417	23035	1.01
Arak	530	14344	0.49
Mashhad	645	77381	2.19
Yazd	431	16751	0.71
Total	4106	224999	1

**Table 3:** Validity of vision screening by health care workers in Iran

**Variable**	**UCVA 20/32 or worse**			
		**Yes**	**No**	**Total**
UCVA 20/32 or worse	Yes	132	257	389
	No	214	3472	3686
Total		346	3729	4075
Sensitivity			38.15 (95%CI: 33.01 to 43.50)	
Specificity			93.11 (95%CI: 92.25 to 93.90)	
Positive Likelihood Ratio			5.54 (95%CI: 4.63 to 6.62)	
Negative Likelihood Ratio			0.66 (95%CI: 0.61 to 0.72)	
Disease prevalence			8.49 (95%CI: 7.65 to 9.39)	
Positive Predictive Value			33.93 (95%CI: 29.24 to 38.88)	
Negative Predictive Value			94.19 (95%CI: 93.39 to 94.93)	

**Fig. 2:**
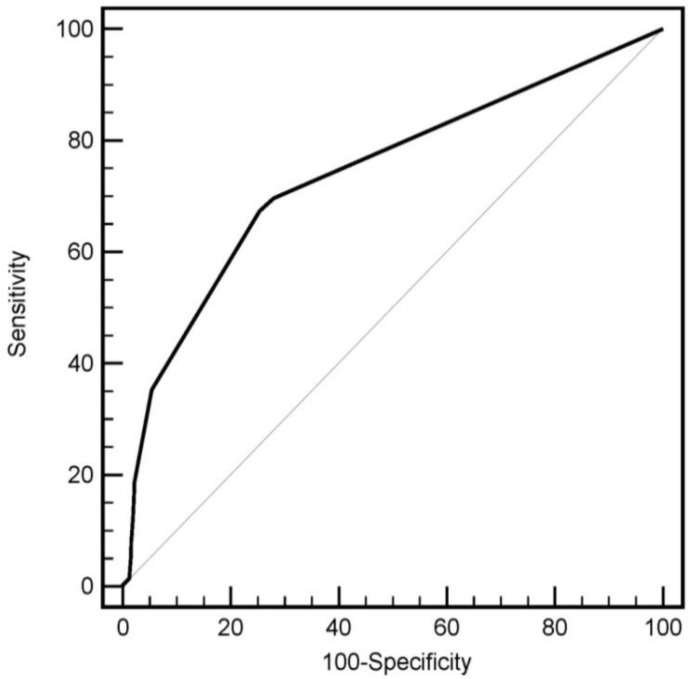
Roc curve: use of uncorrected visual acuity (logmar) by health care screeners to detect visual acuity worse than 20/25 by optometrist

Table 4 presents results in boys and girls. Only the positive predictive value was higher in boys and other indices are not very different between the two groups. Fig. 2 illustrates the ROC curve chart for uncorrected vision testing by health workers compared to optometrists; the area under the ROC curve was 0.741, which significantly differs from 0.5 (*P*<0.001). Based on the Youden’s index, the best-associated criterion was an uncorrected visual acuity more than 0.05 Log-MAR with 67.3% sensitivity and 74.7% specificity.

**Table 4:** Sensitivity of vision screening by public health care workers according to gender

**Variable**	**Male**		**Female**	
	**%**	**95% CI**	**%**	**95% CI**
Sensitivity	39.13	31.55 to 47.12	37.50	30.49 to 44.92
Specificity	93.92	92.68 to 95.00	92.42	91.18 to 93.54
Positive Likelihood Ratio	6.43	4.92 to 8.41	4.95	3.89 to 6.29
Negative Likelihood Ratio	0.65	0.57 to 0.73	0.68	0.60 to 0.76
Disease prevalence	8.61	7.37 to 9.97	8.36	7.23 to 9.59
Positive Predictive Value	37.72	30.35 to 45.54	31.08	25.04 to 37.63
Negative Predictive Value	94.25	93.04 to 95.31	94.19	93.07 to 95.18

## Discussion

In this report, we made a thorough assessment of the validity of the school entry vision screening in Iran. Some previous studies have examined the validity of preschool exams in certain cities (7). Nonetheless, this study has many strong points compared to previous studies, which include random sampling, larger sample size, obtaining results generalized to the entire country, more detailed report and suggesting diagnostic cut points. Our findings indicated a sensitivity rate of 38.15% for screening based on a visual acuity equal to or worse than 20/32. A previous study, conducted in Mashhad, reported a rate of 37.5% (7).

Considering the long history of the screening program, we assumed examiners would be better experienced and expected to see a higher sensitivity rate, but the rate in our study is lower than that in other countries (Table 5) (13, 14). A notable point in this finding is the false negative results. Throughout the country, more than 60% of first graders who have visual acuity equal to or worse than 20/32 is not identified through the screening program and this is while their parents will not seek vision testing because their children have already been examined at school. The sensitivity of the tests in Iran is much lower than that in other countries (Table 5). The reported rate in a country like Sweden is also 33% (15), however, study participants in Sweden were three yr old children and their responses are less valid as well. In another study concerning vision screening in Iranian children, the sensitivity rate was about 74.5% that is significantly higher than our study. This considerable difference can be attributed to the examiners and a final cut point of 20/30 compared to 20/25 in our study (12). Covering a wide range of diseases can lead to reduced sensitivity but in our study, we only focused on two diseases, and thus, the low sensitivity rate is by no means negligible (16). In some parts of the world, objective instruments are used in addition to uncorrected vision testing. Therefore, the current examinations performed in Iran might require a change of method and proper training of the examiners. In addition to their ease of use, objective methods have shown high levels of accuracy in identifying cases with refractive errors and amblyopia without sacrificing the sensitivity of the test or any need for cycloplegia (17, 18). In addition, the instruments have high positive predictive values and can be used for screening purposes by a lay person (19).

**Table 5:** Validity of vision screening in different studies

**Reference**	**Sample Size**	**Age (yr)**	**Sensitivity**	**Specificity**	**PPV**	**NPV**
(27)	949	6–59 Months	50	98.5	57.5	94.4
(28)	51	3–5 Months	83	68	68	83
(13)	112	6–48 Months	82.8	61.8	68.2	48.1
(29)	196	5 Yr	60	91		
(30)	1260	2–5 Yr	91	91		
(15)	400	3 Yr	33	85	9.5	96
(14)	1180	3 Yr	90.9	93.8		
(31)	1218	9–36 Months	37–87	93–99	19–69	96–100
(32)	404	3 Yr	80	58		
(33)	292	4–6 Yr	50	98.9	63	
(34)	122	6 Months To 5 Yr	97.3	80.8	70.6	98.4
(35)	89	Under 4 Yr	53.1	38.5	32.6	
(36)	170	Under 5 Yr	80–88	41–58		
(37)	336	3.5–4.5 Yr	41–95	73–92		
(7)	1163	7–15 Yr	37.5	92	25	94
(38)	2158	7–15 Yr	25	96.9	13.6	98.4

PPV: Positive predictive value

NPV: Negative predictive value

In our study, the predictive value of uncorrected vision testing for screening was 33.93%. The predictive value in our study is lower than that in most previous studies. This is while in some regions or even our neighboring countries such as Oman, rates as high as 99.1% and 99.6% have been reported in Batinaand Dahiram, respectively (20). Unlike other reports, which have high false positive rates, the rate was quite low in our study (21, 22). Positive predictive value correlated inversely with the false positive rate. In other words, a diagnostic test with a low predictive value has a high rate of false positive cases among the screening population. False positive cases, which have no particular problem, are then referred to an ophthalmologist or optometrist for further care and follow up, and this imposes surplus expenses on the health system. On the other hand, parents’ anxiety and concern about their children’s visual health may not be resolved after their follow-up visit with an ophthalmologist or optometrist. Therefore, to improve the predictive value in low-prevalence areas, supplementary tests such as refraction need to be used to identify and refer children with vision problems (23, 24).

Cost is one of the important factors in decision making about screening programs. However, since the main objective of these programs is timely identification and treatment of vision disorders, one must note the efficacy and accuracy as well. These screenings are very cost-effective in Iran, however, employing optometrists can increase the program effectiveness but also the costs (12, 25). Throughout Iran, non-optometrist personnel conduct both screenings for preschoolers and first graders. While, optometrists can improve the sensitivity of the screening test, they have an uneven distribution throughout the nation, therefore, enhancing the knowledge of teachers and other school personnel, as well as their training, is another solution for reducing positive and negative false results (26).

On the other hand, in designing screening programs, we can improve sensitivity rates through other approaches such as changing the cut point. The best cut point for uncorrected visual acuity that provided the highest sensitivity and specificity rates was 0.1. In other words, schoolchildren with vision equal to or worse than 20/25 need to be referred to an ophthalmologist or optometrist. While, this cut point can lead to a higher number of false positive results compared to the previous one, the advantage is that false negative cases will be minimized. Therefore, we recommend using supplementary methods such as objective refraction to reduce the false positive rate. Overall, our findings revealed low validity and high false negative rates for first graders’ vision screening tests performed early in every school year, and this leads to negligence of children with vision problems and imposition of financial burden to the government because most parents rely on test results. Therefore, one priority for the health system is to increase the sensitivity and specificity of these tests. The validity of the tests can be improved by training the examiners of the screening program, using objective instruments, and supplementary methods for measure indices other than visual acuity.

This study had certain strengths and weaknesses mentioned. The most notable strong points include assessment of the status of the school entry vision-screening program through cluster sampling from the entire country and the large sample size. Limitations include lack of access to follow-up information regarding those who had screened positive by health care workers and lack of sufficient data for assessing the cost-effectiveness screening by optometrists.

## Conclusion

The validity of the school entry vision-screening program in Iran is low. To minimize false negative rates, conducting additional examinations such as refraction and near visual acuity measurement, as well as further training of health care screeners should be considered.

## Ethical considerations

Ethical issues (Including plagiarism, informed consent, misconduct, data fabrication and/or falsification, double publication and/or submission, redundancy, etc.) have been completely observed by the authors.
